# Root-Knot Nematode Resistance in *Gossypium hirsutum* Determined by a Constitutive Defense-Response Transcriptional Program Avoiding a Fitness Penalty

**DOI:** 10.3389/fpls.2022.858313

**Published:** 2022-04-13

**Authors:** Jonathan Odilón Ojeda-Rivera, Mauricio Ulloa, Philip A. Roberts, Pratibha Kottapalli, Congli Wang, Héctor-Rogelio Nájera-González, Paxton Payton, Damar Lopez-Arredondo, Luis Herrera-Estrella

**Affiliations:** ^1^Institute of Genomics for Crop Abiotic Stress Tolerance, Department of Plant and Soil Science, Texas Tech University, Lubbock, TX, United States; ^2^USDA-ARS, PA, CSRL, Plant Stress and Germplasm Development Research, Lubbock, TX, United States; ^3^Department of Nematology, University of California, Riverside, Riverside, CA, United States; ^4^Unidad de Genomica Avanzada/Langebio, Centro de Investigacion y de Estudios Avanzados, Irapuato, Mexico

**Keywords:** *Gossypium hirsutum*, *Meloidogyne incognita*, RNA-seq, defense response, jasmonic acid, salicylic acid

## Abstract

Cotton (*Gossypium* spp.) is the most important renewable source of natural textile fiber and one of the most cultivated crops around the world. Plant-parasitic nematode infestations, such as the southern Root-Knot Nematode (RKN) *Meloidogyne incognita*, represent a threat to cotton production worldwide. Host-plant resistance is a highly effective strategy to manage RKN; however, the underlying molecular mechanisms of RKN-resistance remain largely unknown. In this study, we harness the differences in RKN-resistance between a susceptible (Acala SJ-2, SJ2), a moderately resistant (Upland Wild Mexico Jack Jones, WMJJ), and a resistant (Acala NemX) cotton entries, to perform genome-wide comparative analysis of the root transcriptional response to *M. incognita* infection. RNA-seq data suggest that RKN-resistance is determined by a constitutive state of defense transcriptional behavior that prevails in the roots of the NemX cultivar. Gene ontology and protein homology analyses indicate that the root transcriptional landscape in response to RKN-infection is enriched for responses related to jasmonic and salicylic acid, two key phytohormones in plant defense responses. These responses are constitutively activated in NemX and correlate with elevated levels of these two hormones while avoiding a fitness penalty. We show that the expression of cotton genes coding for disease resistance and receptor proteins linked to RKN-resistance and perception in plants, is enhanced in the roots of RKN-resistant NemX. Members of the later gene families, located in the confidence interval of a previously identified QTL associated with RKN resistance, represent promising candidates that might facilitate introduction of RKN-resistance into valuable commercial varieties of cotton. Our study provides novel insights into the molecular mechanisms that underlie RKN resistance in cotton.

## Introduction

Cotton (*Gossypium* spp.) is one of the most widely cultivated crops worldwide. The genus *Gossypium* includes more than 45 diploids and 7 allotetraploid species, of which Upland and non-Upland Acala [*G. hirsutum* L. (*Gh*)] and Pima [*G. barbadense* (*Gb*)] account for 95 and 2% of the global annual cotton crop production, respectively (Chen et al., 2007). *Gh* and *Gb* are allotetraploid cotton lineages that were independently domesticated for their seed fiber and possess an AD genome that resulted from polyploidization between A-genome African (*G. arboreum*-like) species and D-genome American (*G. raimondii*-like) species (Wendel and Grover, 2015). More than 33 million hectares of cotton are cultivated annually around the world, which highlights cotton as the leading textile fiber-producing crop. Cotton accounts for up to 25% of the total fiber used worldwide and represents an industry with an economic impact of approximately 500 billion US dollars (Chen et al., 2007; Zhang et al., 2014).

Plant-parasitic nematode infestations result in an average annual worldwide loss of up to 14% of crop production worldwide, representing economic losses of more than $100 billion US dollars per year (Sasser and Freckman, 1987; Mitkowski and Abawi, 2003; Williamson and Kumar, 2006). Root infestation by parasitic nematodes is a critical constraint for cotton production. Nematode damage caused by the southern Root Knot Nematode (RKN), *Meloidogyne incognita* Kofoid and White (Chitwood), is a biological threat that has spread rapidly in over 143 countries around the world (Bebber et al., 2014). *M. incognita* invades the root tissues by both mechanical piercing and through secretion of a diverse array of cell wall degrading enzymes (Abad et al., 2008; Opperman et al., 2008; Danchin et al., 2010). The feeding sites of this nematode consist of hypertrophied, multinucleated, and metabolically active feeding cells (giant cells) enclosed in root galls; these cells do not undergo division and serve as a nutrient reservoir for the parasite (Mitkowski and Abawi, 2003). The infested tissues reduce transport of water and nutrients from the root system into the aerial part of the plant, resulting in stunted growth and lower yields (Williamson and Hussey, 1996). Consequently, RKN-related losses are estimated at about 10% reduction in cotton yield (Lu et al., 2014). Several studies showed that there is a detrimental and synergistic role of RKN in the *Fusarium* wilt syndrome of cotton, caused by *Fusarium oxysporum* sp. f. *vasinfectum* (FOV). Together with FOV, *M. incognita* forms a disease complex (RKN-FOV) which is a combination of nematode and fungal infection that results in an increased incidence and severity of *Fusarium* wilt disease (Abawi and Chen, 2015).

Given the multiple negative repercussions of RKN infestation in agriculture, nematode resistance has been given special attention in breeding programs including those for tomato, common bean, soybean and cotton among other crop species (Roberts, 1992; Ulloa et al., 2010). Effective resistance is available for improving cotton cultivar performance (Starr et al., 2010). There are multiple germplasm sources of RKN resistance that have been utilized in *Gh* cotton breeding programs. One of these resistant sources is the ‘Acala NemX’ (NemX) cultivar (Ogallo et al., 1997; Wang et al., 2006a; Roberts and Ulloa, 2010). Another source of resistance gene is found on chromosome D02 of the Wild Mexico Jack Jones line, which contributes to suppression of nematode egg production (Gutiérrez et al., 2010; He et al., 2014; Kumar et al., 2016). Our previous studies have mapped the RKN-resistance of NemX to two regions located in chromosomes A11 and D11 (Ulloa et al., 2010; Wang et al., 2017, 2020), in which genes encoding CC-NB-LRR and TIR-NBL-LRR R proteins involved in the activation of plant defense responses were found (Spoel and Dong, 2012). While these studies establish that resistance is common in cotton, the underlying functional mechanisms for disease resistance are not known.

Host-plant resistance is a highly effective strategy to manage root-knot nematode (*Meloidogyne* spp.) damage in crops. In the case of cotton, NemX was released by the California Planting Cotton Seed Distributors (CPCSD) 30 years ago as a cultivar with high resistance to RKN. The RKN-resistance present in NemX is highly effective in protecting against the effects of RKN infection and has been used as a source of nematode-resistance in breeding and genetic studies (Roberts and Ulloa, 2010; Ulloa et al., 2010; Wang et al., 2020). NemX has a higher yield under RKN and RKN-FOV infection than other cultivars, and increases the rotational value of crops (Ogallo et al., 1997). Another *Gh* cultivar that provided nematode-resistance in derived progeny is WMJJ, which presents mild tolerance to RKN and has also been used as a source of nematode-resistance in cotton genetic studies (Gutiérrez et al., 2010; Ulloa et al., 2010). Acala SJ-2 (SJ2), another Acala-type cultivar released by the USDA-ARS a few decades ago, contrary to NemX, is susceptible to RKN.

Since cotton cultivars SJ2, WMJJ, and NemX present low, intermediate, and high levels of resistance to RKN infestation, respectively, they represent excellent models to get insights into the genetic basis and the transcriptional regulation of the RKN-response in resistant and susceptible cotton plants. In this study, we harness the differences in RKN-resistance among the *Gh* cultivars SJ2 (susceptible to RKN), WMJJ (moderately resistant to RKN), and NemX (resistant to RKN), to get insights into the molecular basis of RKN-resistance in cotton. Our results reveal that constitutive transcription of components of the salicylic and jasmonic acid signaling pathways underlie RKN-resistance in cotton, and we identify candidate genes potentially involved in the activation of plant defense response to RKN.

## Materials and Methods

### Plant Materials

Cotton cultivar Acala NemX was selected for resistance, Acala SJ-2 for susceptibility, and WWJJ (Wild Mexico Jack Jones) was selected for mildly tolerance to RKN infection.

### RKN Inoculum and Infection Process

To evaluate RKN resistance, three-week-old seedlings were inoculated with approximately 50,000 eggs of *M. incognita* race 3 (isolate Project 77, originating from an Upland cotton field in San Joaquin Valley, CA). Air temperatures in the greenhouse were maintained between 28 and 35°C during the day and 24°C at night. Cotton plants were evaluated for resistance reaction 60 days after inoculation for cultivars NemX, SJ2, WMJJ. A 0–10 root galling index (GI) rating scale (modified from Bridge and Page, 1980) was used to evaluate resistance reaction to nematodes. Briefly, this scale consists on a RKN-rating chart that ranges from 0 = no galls, 1 = few small galls, 2 = small galls with less than 10% of roots infected, 3 = 10–30% of roots infected, main roots clean, 4 = 31–40% of roots infected, 5 = 51–60% of roots infected, knotting on parts of main roots, 6 = 61–70% of roots infected, knotting on main roots, 7 = 71–80% of roots infected, majority of main roots knotted, 8 = 81–100% of roots infected, all main roots knotted, 9 = all roots severely knotted and plant usually dying, 10 = all roots severely knotted with diminished root system and plant usually dead (Bridge and Page, 1980). Plants were classified as resistant or susceptible based on the susceptible and resistant parent phenotypes in each test as described in Wang et al. (2006a) and Ulloa et al. (2010). Cotton cultivars were grown in a complete randomized design and with six plant-replications per cotton genotypes. Phenotypic GI data were subjected to one-way analysis of variance (ANOVA). Fisher’s Protected LSD test was used to compare the treatment means using SAS (SAS, Ver. 9.1.3; SAS Institute, Cary, NC, United States).

For RNA-seq, the roots of 12-day-old seedlings of three genotypes NemX (resistant), SJ2 (susceptible) and WMJJ (moderately resistant) were infected with 5,000 J2/plant RKN or mocked-inoculated with water only (non-inoculated controls) as detailed in Ulloa et al. (2010). The disease progression was noted for 10 days and infected roots from 23-day-old plants were collected from each of the six plants per genotype per treatment (see Table 1), flash-frozen in liquid nitrogen, and stored at −80°C until RNA extraction.

**Table 1:** Alignment statistics and information about the 18 RNA-seq libraries generated in this study. All libraries were sequenced using Illumina technology and aligned to *Gossypium hirsutum* TM-1 UTx v2.1 genome (Chen et al., 2020).

Library name	Genotype	Treatment	Biological replicate	Number of reads	Kallisto pseudo-aligned reads	% of pseudo-alignment	Type of library
SJ2_ctl1	SJ2	Control	1	5,074,040	4,316,080	85.06	Paired-end
SJ2_ctl2	SJ2	Control	2	5,365,176	4,744,066	88.42	Paired-end
SJ2_ctl3	SJ2	control	3	8,401,787	7,321,725	87.14	Paired-end
SJ2_rkn1	SJ2	RKN-infested	1	5,672,349	5,098,209	89.88	Paired-end
SJ2_rkn2	SJ2	RKN-infested	2	3,146,441	2,741,297	87.12	Paired-end
SJ2_rkn3	SJ2	RKN-infested	3	10,999,880	9,472,493	86.11	Paired-end
WMJJ_ctl1	WMJJ	Control	1	4,769,048	3,359,862	70.45	Paired-end
WMJJ_ctl2	WMJJ	Control	2	5,308,861	4,541,480	85.55	Paired-end
WMJJ_ctl3	WMJJ	Control	3	7,860,698	6,479,223	82.43	Paired-end
WMJJ_rkn1	WMJJ	RKN-infested	1	4,978,402	4,370,251	87.78	Paired-end
WMJJ_rkn2	WMJJ	RKN-infested	2	4,086,287	3,593,656	87.94	Paired-end
WMJJ_rkn3	WMJJ	RKN-infested	3	14,771,799	12,594,074	85.26	Paired-end
NemX_ctl1	NemX	Control	1	4,699,626	4,118,656	87.64	Paired-end
NemX_ctl2	NemX	Control	2	4,881,368	4,461,912	91.41	Paired-end
NemX_ctl2	NemX	Control	3	8,421,811	7,208,028	85.59	Paired-end
NemX_rkn1	NemX	RKN-infested	1	7,776,606	6,729,422	86.53	Paired-end
NemX_rkn2	NemX	RKN-infested	2	4,475,985	4,027,422	89.98	Paired-end
NemX_rkn3	NemX	RKN-infested	3	4,468,432	4,051,932	90.68	Paired-end

### RNA Isolation, Quantification, and Quality

Root samples from each genotype were grounded in liquid nitrogen, and total RNA was isolated from three biological replicates using Spectrum^™^ Plant Total RNA kit (Sigma-Aldrich, St. Louis, MO, United States) following the manufacturer’s instructions. The yield and purity of RNA were analyzed with a ND-1000 Spectrophotometer (Nano Drop Technology, Wilmington, DE, United States). Only RNA samples with 1.8–2.2 ratio of absorbance 260/280 nm were used for analysis.

### cDNA Library Preparation and Sequencing

For each genotype, 2 μg of RNA from each of the three biological replicates was used for cDNA library preparation. The cDNA libraries were prepared following the TruSeq RNA Sample Preparation v2 low sample (LS) protocol guide (Illumina Inc., San Diego, CA United States) as described in Kottapalli et al. (2016) manually. One-third of the samples were prepared using TruSeq stranded mRNA on the Neoprep^™^ following manufacturer’s instructions.

Each of the 10 nM cDNA libraries was diluted to 4 nM with hybridization buffer and multiplexed (four samples were pooled). Pooled cDNA libraries were denatured with NaOH and normalized to 10 nM concentration. A final concentration of 5.4 pM was loaded onto the MiSeq Reagent cartridge (MiSeq Reagent Kit v2 300 cycles, Illumina Inc., San Diego, CA United States). For the sequencing on the HiSeq 2,500 platform, we pooled equimolar concentrations of 24 samples into one single Rapid run of 2 lanes. A final concentration of 6.3pM was loaded onto the HiSeq2500 sequencer (HiSeq 2500, Illumina Inc., San Diego, CA, United States). Paired end sequencing with 150 bp paired-end reads was performed on a HiSeq Rapid v2 flow cell from Illumina as per manufacturer’s protocols.

### Data Analysis

We first performed quality assessment of the resulting reads from the Illumina platform using FastQC (version 0.11.9)^1^ and processed sequencing libraries using Trimmomatic [version 0.39; (Bolger et al., 2014)] to remove adapter read sequences. We then quantified gene expression using the pseudo-alignment RNA-seq quantification program kallisto version 0.46.1 (Bray et al., 2016). The *G. hirsutum* TM-1 UTx v2.1 genome release (Chen et al., 2020) was used as a reference genome to align the reads. We then integrated transcript-level abundances from kallisto to count-based statistical analysis in edgeR (version 3.13; Robinson et al., 2010) and DEseq2 (release 3.13; (Love et al., 2014) using the tximport package (Soneson et al., 2016). We determined differential gene expression profiles in response to RKN-infection (rkn vs. ctl) using a 1 > logFC<−1 threshold and false discovery rate threshold (FDR <0.01). We performed analysis of intersections between the sets of differentially expressed genes among genotypes using the UpSetR package version 1.4 (Conway et al., 2017).

To perform Gene Ontology (GO) analysis, we first developed a GO functional annotation of the TM-1 UTx v2.1 genome which was assigned using a simplified version of the maize-GAMER pipeline (Wimalanathan et al., 2018). Briefly, annotations were assigned as the GO annotations of the blastp reciprocal best hits versus Araport11 (Cheng et al., 2017) and UniProt (Bateman et al., 2021), Swiss-Prot proteins from nine plant species (*Glycine max, Oryza sativa subsp. japonica, Populus trichocarpa, Solanum lycopersicum, Sorghum bicolor, Vitis vinifera, Brachypodium distachyon, Physcomitrium patens, and Chlamydomonas reinhardtii*), the GO annotations from an Interproscan [v5.48.83; (Blum et al., 2021)] analysis, and the GO annotations from the PANNZER2 (Törönen et al., 2018) functional annotation webserver with a PPV value of at least 0.5. Analyses were collated into a non-redundant gtf file and used for GO enrichment analyses. The GO annotation is available in Supplementary Material. GO enrichment analysis was performed using the clusterProfiler package (Wu et al., 2021). Heatmaps were prepared using the ComplexHeatmaps package (Gu et al., 2016) and z-score was used as a measure of relative expression. We took advantage of the protein-homology database of *G. hirsutum* TM-1 UTx v2.1 genome (Chen et al., 2020)^2^ to identify homologous proteins in *Arabidopsis thaliana*. Homologous proteins in *A. thaliana* were determined using the blastx algorithm against the Arabidopsis proteins (Araport11) with an expectation value cutoff less than 1^e-6^.

### Quantification of Phytohormone Levels by UHPLC–MS

To determine jasmonic and salicylic acid levels, leaf tissue from three 50-day-old SJ2 and NemX cotton plants was collected and frozen immediately using liquid nitrogen. Each cotton plant was accounted as one biological replicate (*n* = 3). Salicylic and jasmonic acid levels were assessed using liquid chromatography (UHPLC) coupled to mass spectrometry (MS) detection. Extraction and quantification were performed according to Segarra et al. (2006) with slight modifications. Briefly, tissue was powdered in liquid nitrogen and freeze dried for 24 h. Metabolite extraction was performed by adding 1 ml of methanol:water mixture 90:10 with 1% formic acid (FA) to 300 mg of tissue. After extraction, the solvent was concentrated four times in a rotary evaporator (Labconco, United States) and directly injected to the UHPLC–MS system (Vanquish Flex UHPLC coupled to Orbitrap Exploris 240 from Thermo Electron, North America). Quantification was performed using external standard methodology in SRM mode. Transition monitored for salicylic acid was 137.0244 → 93.034, while for jasmonic acid the 209.1183 → 59.013 using negative ionization mode. Chromatographic separation was achieved in a Reversed Phase C18 column Acclaim VANQUISH 150 × 2.1 mm, with 2.2 μm particle size, using water 0.1% FA and methanol 0.1% FA as mobile phase A and B, respectively. Gradient method started with 5% B 1–3.5 min followed by 5–100% B from 3.5 to 13 min, maintaining 100% B for 1 min and then re-equilibrating the column for 2.5 min to 5% B. Retention time for salicylic acid was 12.57 min and for jasmonic acid was 13.21 min. Standards were acquired from Sigma-Aldrich (St. Louis, MO, United States).

## Results

### RKN Resistance Evaluation Corroborated Differential RKN Resistance Among *Gossypium hirsutum* SJ2, WMJJ, and NemX Genotypes

**Figure 1:**
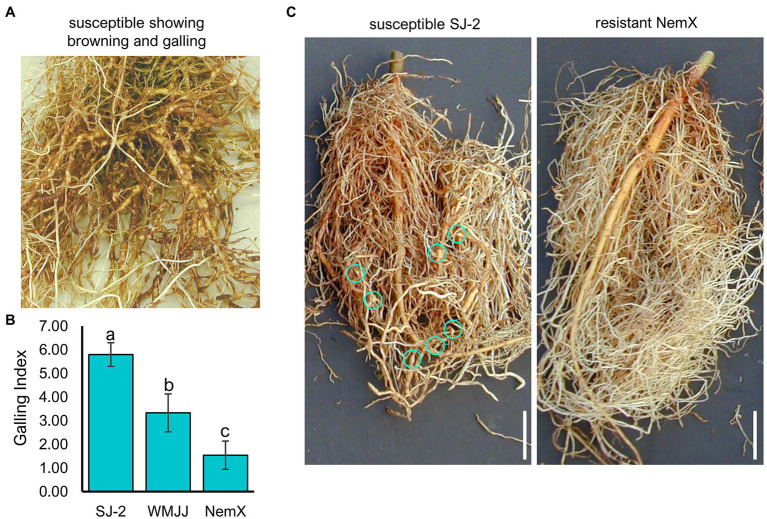
The cotton cultivars SJ2, WMJJ and NemX present low, mild and high resistance to RKN infection, respectively. **(A)** Severe symptoms of nematode infection in the root system of a susceptible plant. **(B)** Distribution of mean Galling Index (GI) for the cotton cultivars Acala NemX (NemX, RKN-resistant), Acala SJ-2 (SJ2, RKN-susceptible), Wild Mexico Jack Jones (WMJJ, moderately RKN-resistant). Different letters represent difference in susceptibility to RKN (*Meloidogyne incognita* – RKN). GI: 0–10 scale on Y-axis. **(C)** Images depicting RKN-infested roots of NemX and SJ2 evaluated at 60 days. Blue circles indicate the presence of root galls. Scale bar: 5 cm.

Severe symptoms of nematode infection include browning of the root system, caused by cell death, and the formation of root galls (Figure 1A). Differences in root galling index (GI) were observed between SJ2, WMJJ, and NemX (*p >* 0.05; Figure 1B). The average GI on resistant NemX and susceptible SJ2 were 1.6 and 5.8, respectively, while WMJJ was moderately resistant with GI of 3.3. Previous reports (Wang et al., 2006a; Ulloa et al., 2010) have demonstrated that root GI is highly correlated (*r =* 0.79) with RKN eggs/g of root in tests with cultivars SJ2, NemX, and Pima S-7 (*G. barbadense* L.) and derived populations. In general, susceptible SJ2 supported larger numbers of RKN eggs/g of root (12,431 eggs/g) than resistant NemX (518 eggs/g) and presented more evident symptoms of nematode infection in the root system including root browning and presence of root galls (Ulloa et al., 2010; Figure 1C).

### Differential Expression Profiling of SJ2, WMJJ, and NemX Revealed a Constitutive RKN-Defensive Transcriptional Behavior in NemX Roots

To dissect the molecular responses to RKN infection in cotton roots, we performed RNA-seq profiling of SJ2, WMJJ, and NemX RKN-inoculated and mock-treated plants (see section “Materials and Methods” for details). A total of 18 RNA-seq libraries were produced for the three genotypes (see Materials and Methods, NCBI accession number GSE190503). Libraries were named according to treatment with control abbreviated as ‘ctl’ and RKN-infested abbreviated as ‘rkn’, namely, SJ2_ctl, SJ2_rkn, WMJJ_ctl, WMJJ_rkn, NemX_ctl, and NemX_rkn. Metrics of the different RNA-seq libraries are presented in Table 1.

**Figure 2:**
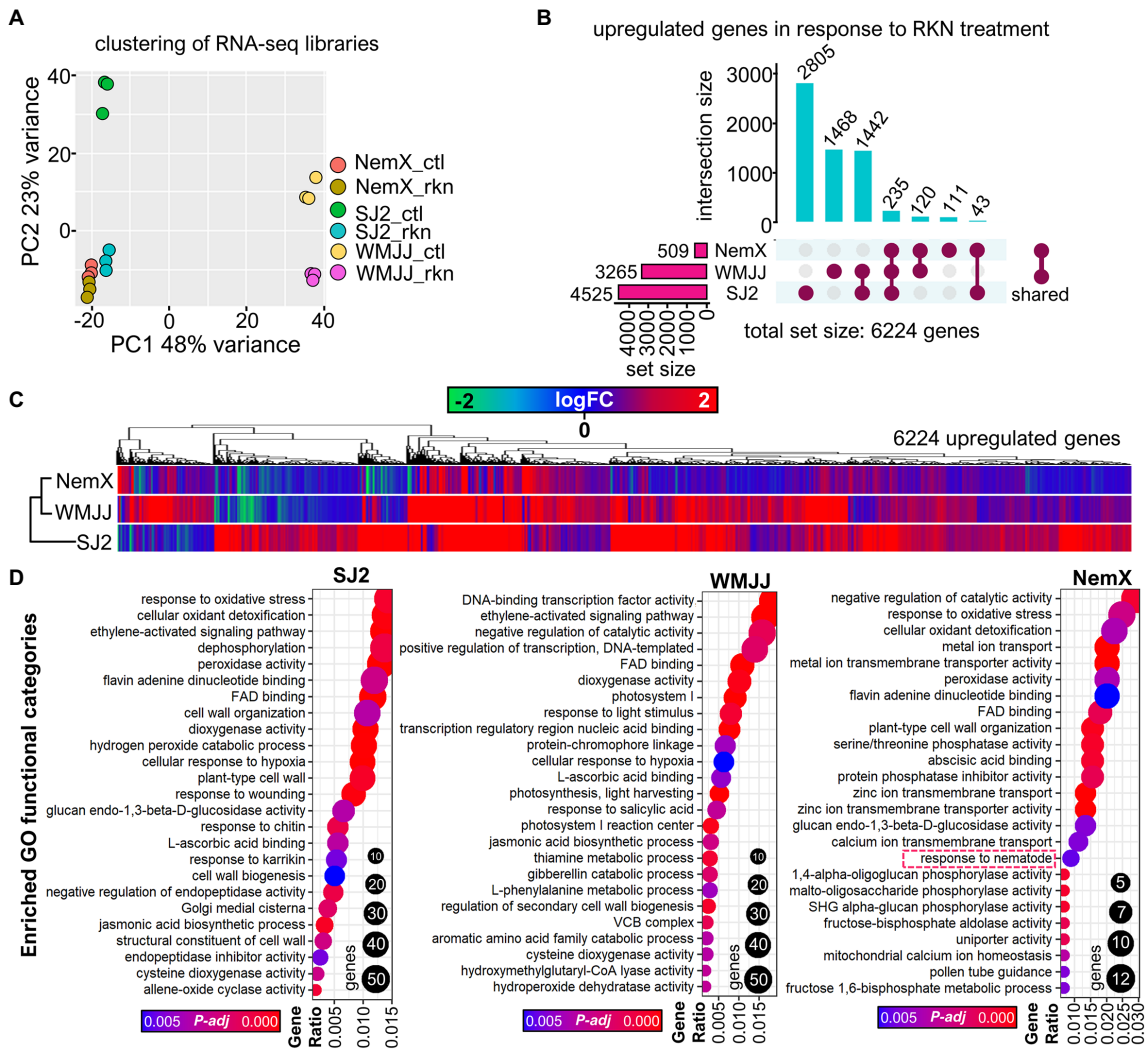
Expression profiling analysis revealed a differential activation of genes and biological processes in response to RKN infection among cotton cultivars SJ2, WMJJ and NemX. **(A)** PCA analysis of sample relationships for each of the RNA-seq libraries of this study. **(B)** Venn analysis of upregulated genes in response to RKN-treatment. **(C)** Heatmap of the upregulation (in logFC units) of the total set of induced genes in SJ2, WMJJ and NemX. **(D)** Gene Ontology (GO) analysis of significantly enriched functional categories (see Supplementary Material).

We first determined gene expression differences among the genotypes and treatments tested (Figure 2). Principal component analysis (PCA; Figure 2A) revealed that samples were clustered by treatment and genotype as expected, which corroborated that the tested experimental conditions were adequate to analyze differential gene expression in response to RKN-infection. Interestingly, the SJ2 and WMJJ libraries of RKN-infected roots (SJ2_rkn and WMJJ_rkn, Figure 2A) clustered differentially in response to both genotype and treatment. However, in the case of the NemX samples (NemX_rkn and NemX_ctl), we unexpectedly found that the samples clustered only according to genotype and not by treatment, suggesting that the NemX plants do not activate major gene expression changes in response to RKN-infection.

We then performed pairwise comparisons between the genotypes and treatments tested to determine differential gene expression (Figures 2B,C; Supplementary Figure S1, Supplementary Material). In SJ2 genotype, which is susceptible to RKN-infection, we discovered 4,525 upregulated genes and 3,722 downregulated genes in response to RKN treatment (8,247 differentially expressed genes in total). In the intermediate-RKN resistant WMJJ cultivar., we determined 3,265 upregulated and 2,920 downregulated genes. In the RKN-resistant NemX, we found out that only 509 and 584 genes were up and downregulated, respectively, in response to RKN-infestation (Figures 2B,C; Supplementary Figure S1). These data show that there is less activation of differential gene expression in response to RKN infestation in WMJJ than in SJ2 (6,184 genes, ~75% of the total differentially expressed genes in SJ2) and that there is a significantly more discrete transcriptional response to RKN-infestation in NemX in comparison with both SJ2 and WMJJ (1,093 genes, ~13% of SJ2 and ~15 of WMJJ). Using an UpSet analysis to determine the sets of genes that are common or uniquely upregulated across the different cultivars or genotypes, we observed that there is a common set of 235 genes that are activated in all the genotypes in response to RKN infection; a set of 43 genes induced in both SJ2 and NemX; a set of 1,442 genes that are activated in SJ2 and WMJJ; and a set of 120 genes that are only activated by NemX and WMJJ (Figure 2B). Genes with increased transcript levels specific for each cultivar were 111, 1,468 and 2,805 in NemX, WMJJ and SJ2, respectively (Figure 2B).

### GO Profiling of SJ2, WMJJ, and NemX Cotton Cultivars in Response to RKN-Infection

To further characterize the biological processes that are activated in SJ2, WMJJ and NemX cultivars in response to RKN infection, we carried out Gene Ontology (GO) enrichment analysis of the genes that are induced in the roots of each cultivar in response to RKN-infection (Figure 2D). Diverse GO categories were found to be enriched (adj. value of *p* < 0.05) in each of the cultivars with specific GO profile (Figure 2D and Supplementary Material). Despite the large degree of variation in the GO categories, we did find commonly enriched GO categories. For instance, we found out that ethylene signaling (GO:0009873 “ethylene-activated signaling pathway”) was activated in the roots of the three genotypes tested in response to RKN-infection. This was expected as ethylene production and signaling has been reported to be triggered by nematode-induced damage (Sato et al., 2019), particularly in the region of the root that is damaged by the parasite (Marhavý et al., 2019). Several categories related to reactive oxygen species (ROS; GO:0006979 response to oxidative stress, GO:0098869 cellular oxidant detoxification, GO:0042744 hydrogen peroxide catabolic process, GO:0004601 peroxidase activity) were activated in the RKN-infested roots of SJ2 and NemX but not in WMJJ. Oxidative burst is a common immune response of plants to RKN-infection which occurs during the infection process (Sato et al., 2019) and it seems to be a characteristic transcriptional response of Acala-type cultivars as we did not find these ROS-related GO categories to be particularly enriched in the WMJJ genotype (Figure 2D and Supplementary Material). Furthermore, we found enriched GO categories related to jasmonic acid biosynthesis (GO:0009695 jasmonic acid biosynthetic process) and to response to salicylic acid (GO:0009751 response to salicylic acid) in both susceptible SJ2 and mildly resistant WMJJ (Figure 2D; Supplementary Material). However, we did not find these categories, or any other category related to salicylic and jasmonic acid significantly enriched in the genes that are activated in the roots of NemX in response to RKN-infestation (Figure 2D; Supplementary Material). This was unexpected since jasmonic and salicylic acid are two key plant hormones that modulate plant immunity (Pieterse et al., 2012) and NemX is an RKN resistant cultivar.

### Expression Profiling Analysis Revealed Constitutive Activation of Nematode-Related and Defense-Response Related Genes in the NemX and WMJJ RKN-Resistant Cultivars

**Figure 3:**
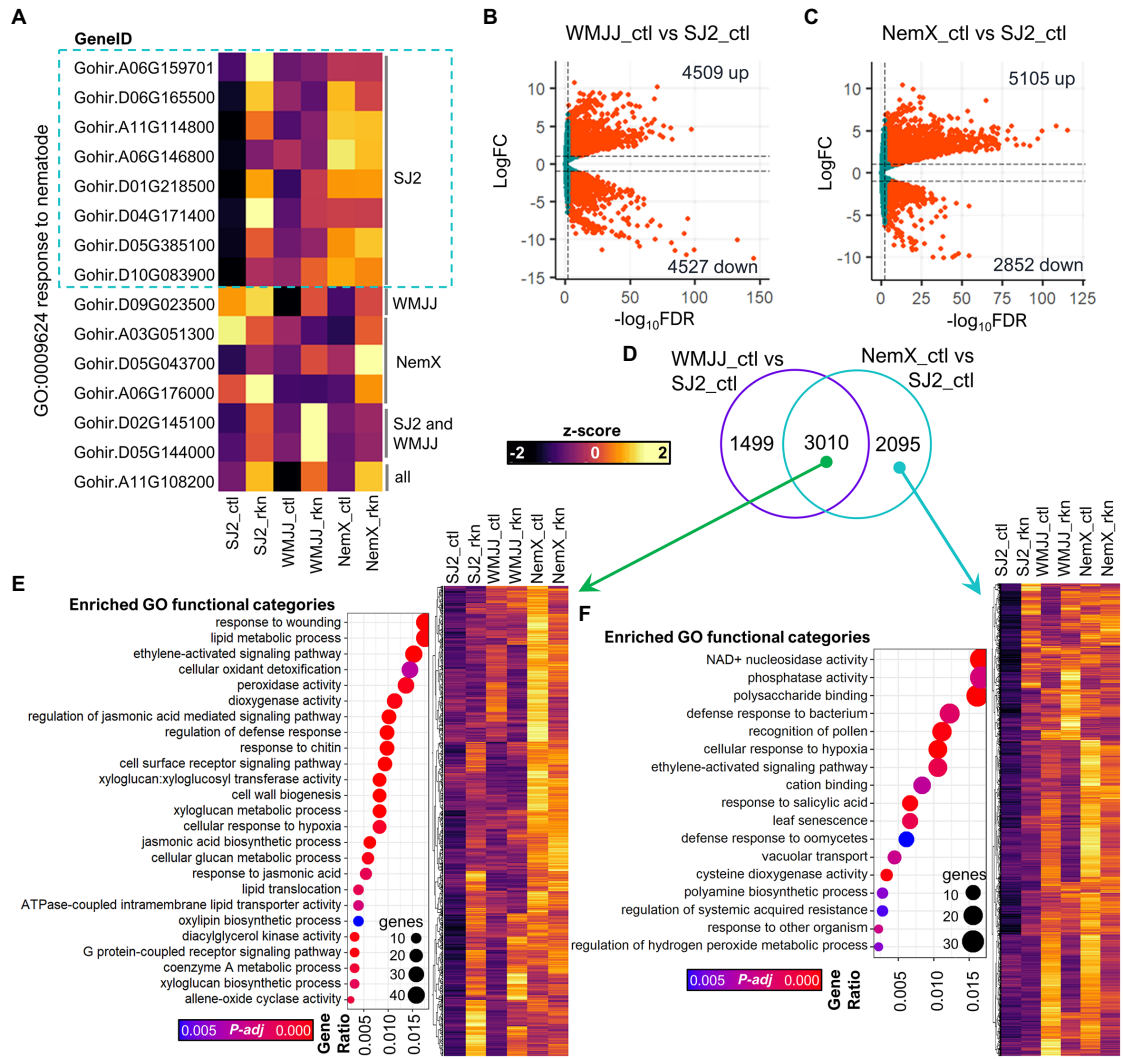
Expression profiling analysis revealed constitutive activation of nematode-related and defense-response related genes in the NemX accession. **(A)** Heatmap of the expression of genes included in the GO:0009624 response to nematode functional category. **(B,C)** Volcano plots of differential basal expression in WMJJ **(B)** and NemX **(C)** cultivars vs. SJ2. **(D)** Venn analysis of the differentially expressed genes presented in **(B,C)**. **(E,F)** GO enrichment analysis of the genes with more basal expression in WMJJ and NemX **(E)** and of those that are specific to NemX **(F)**.

GO analysis indicates that the “response to nematode” (GO: GO0009624) category is significantly enriched in the gene sets that are induced in response to RKN-treatment in the roots of NemX and SJ2, respectively (Figure 2D; Supplementary Material); and this category was found to be more significantly enriched in resistant NemX (adj. value of *p* 0.018) than in SJ2 (adj. value of *p* 0.027) and WMJJ (adj. value of *p* 0.86; not enriched). Because the genes that belong to the “response to nematode” might be of interest to dissect RKN-resistance, we analyzed their expression values among the genotypes and treatments tested (Figure 3A). Interestingly, we discovered that “response to nematode” genes induced in susceptible SJ2 in response to RKN-treatment have a higher basal expression in moderately resistant WMJJ than in SJ2, whereas the highest basal expression was found to be induced in the nematode-resistant NemX (Figure 3A). These data prompted the hypothesis that, rather than activate the expression of RKN-resistance genes, RKN-resistant genotypes present a higher constitutive or basal expression of RKN-resistance related genes. To further test this hypothesis, we performed pairwise comparisons among the root transcriptomic profiles of moderately RKN-resistant WMJJ and RKN-resistant NemX versus susceptible SJ2 under non-infected control conditions, to determine the differences in basal gene expression by genotype (Figures 3B,C). We determined that in WMJJ there are 4,509 genes with higher and 4,527 with lower transcript level respect to SJ2 (Figure 3B). In the case of NemX, we determined that 5,105 genes had higher and 2,852 lower transcript levels with respect to SJ2 (Figure 3C). Interestingly, Venn analysis revealed that WMJJ and NemX have higher transcript levels for 3,010 genes in common with respect to SJ2 under control non-infected conditions (Figure 3D).

Evidence presented in Figures 3B–D supports the notion that there is a wide landscape of basal transcriptional differences between the three genotypes tested that correlate with the degree of RKN resistance. However, it remained unknown whether these genes contribute to biological processes related to RKN-resistance. Therefore, we then performed GO enrichment analysis to get functional insights into the “constitutively expressed” gene sets in RKN-resistant genotypes (Figures 3E,F; Supplementary Figure S2, Supplementary Material). GO enrichment analysis determined that several functional categories related to RKN-responses are significantly enriched for the genes which are upregulated in both WMJJ and NemX with respect to SJ2 (Figure 3E). These categories include “response to wounding” (GO:0009611), “regulation of defense response” (GO:0031347) and “cell surface receptor signaling pathway” (GO:0007166). Furthermore, several categories related to jasmonic acid synthesis and signaling were found to be enriched in this gene set (GO:2000022, GO:0009753, GO:0009695, GO:0031408; Figure 3E; Supplementary Material). In the case of the genes that were upregulated only in WMJJ (1,499 genes) with respect to SJ2, we determined that categories related to terpenoid synthesis, which have been reported to have nematicide activity (Abdel-Rahman et al., 2013), are enriched in this gene set (Supplementary Figure S2; Supplementary Material). In the case of the genes that were upregulated only in NemX (2095 genes) with respect to SJ2, we determined that categories related to defense responses including “defense response to bacterium” (GO:0042742), “response to other organism” (GO:0051707) and “defense response to oomycetes” (GO:0002229) were enriched. Interestingly, several categories related to salicylic acid signaling and acquisition of systemic resistance (GO:0009751, GO:0010112, GO:0009862) were also found to be enriched in the set of genes that possess more basal expression in the roots of NemX than in the roots of SJ2 (Figure 3F). Overall, GO enrichment analysis of the gene sets that have higher basal expression in RKN-resistant genotypes supports the idea that RKN-resistance in the studied cotton cultivars is mediated, at least in part, by an enhanced basal transcription of RKN-resistance-related genes. Of special interest is the fact that the synthesis and signaling of the key plant defense phytohormones jasmonic and salicylic are transcriptionally enhanced in RKN-resistant NemX cultivar.

### Protein Homology Analysis Revealed Basal Expression of Jasmonic Acid Biosynthesis and Signaling Is Upregulated in NemX and WMJJ With Respect to SJ2

**Figure 4:**
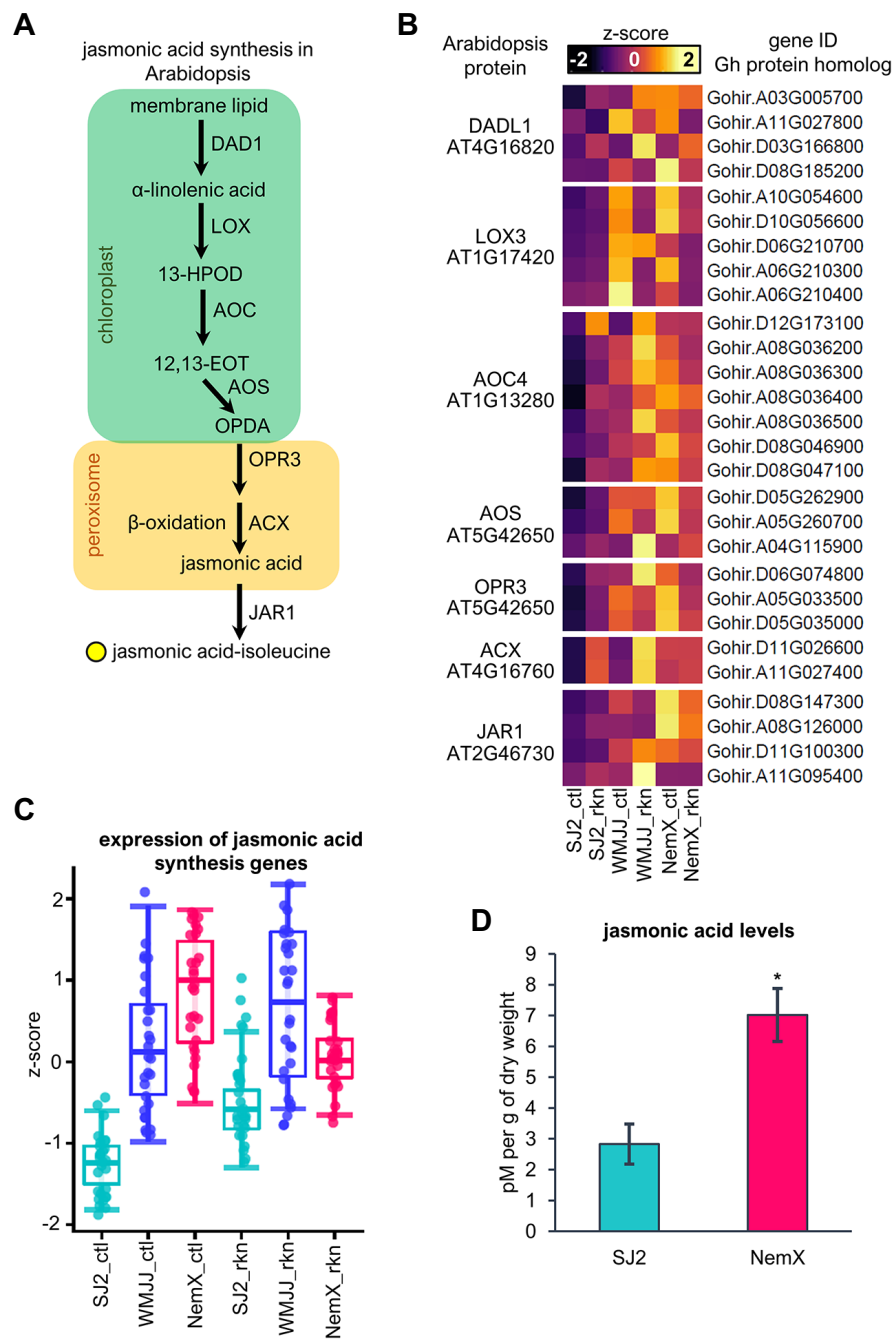
Basal expression of jasmonic acid biosynthesis is upregulated in NemX and WMJJ with respect to SJ2. **(A)** Jasmonic acid synthesis in Arabidopsis. **(B)** Heatmap of the expression of *Gh* genes coding for orthologous proteins of Arabidopsis. **(C)** Boxplot analysis of the expression levels of jasmonic acid synthesis genes presented in **(B)**. **(D)** Quantification of jasmonic acid in SJ2 and NemX cotton plants using UHPLC coupled to MS. Jasmonic acid levels were determined in picomoles (pM) per gram of dry weight. Student’s *t*-test was performed to determine statistically significant differences (*n* = 3) which are indicated with an asterisk ( *p* <0.05).

The synthesis of jasmonic acid starts in the chloroplast where membrane phospholipids are hydrolyzed by a phospholipase that catalyzes the formation of α-linolenic acid from membrane galactolipids; α-linolenic acid is further processed by a lipoxygenase (LOX), an allene oxide synthase (AOS), and an allene oxide cyclase (AOC) to produce 12-oxo-phytodienoic acid (OPDA). OPDA is transported into the peroxisome where it is converted to jasmonic acid by OPDA-reductase and three consecutive cycles of β-oxidation by an acyl-CoA-oxidase (ACX; Figure 4A). Ultimately, jasmonic acid is conjugated to isoleucine (ja-isoleucine), which is the biologically active form of jasmonic acid (Wasternack and Song, 2017; Figure 4A). To further characterize the upregulation of jasmonic acid synthesis in RKN-resistant cotton genotypes, we decided to analyze the expression of genes that are more expressed in RKN-resistant NemX and moderately resistant WMJJ than in susceptible SJ2 that code orthologs of *Arabidopsis* enzymes that are involved in jasmonic acid synthesis (Figure 4B), because jasmonic acid synthesis has been thoroughly characterized in *Arabidopsis* (see Wasternack and Song, 2017 for review).

In *Arabidopsis*, membrane lipid hydrolysis is catalyzed by the enzymes encoded by the genes *DEFECTIVE IN ANTHER DEHISCENCE 1 (AtDAD1)* and its paralog *AtDAD1-LIKE* (Ishiguro et al., 2001; Ruduś et al., 2014) for which we found 4 cotton (*Gh*) genes encoding *Gh*DAD1-LIKE protein orthologs (Figure 4B). In the particular case of nematode infection in *Arabidopsis*, further oxidation of α-linolenic acid is increased and carried out by the enzymes encoded by the genes *AtLOX3* and *AtLOX4* (Ozalvo et al., 2014) for which we found 5 *Gh* genes encoding *Gh*LOX3 protein orthologs. The *Arabidopsis* genome has four genes that code for AtAOCs of which *AtAOC4* is predominantly expressed in roots (Stenzel et al., 2012); we found 7 *Gh* genes encoding for *Gh*AOC4 orthologous proteins. The main allene oxide synthase of *Arabidopsis* is encoded by *AtAOS* (Laudert and Weiler, 1998) for which we found 3 *Gh* genes encoding AtAOS orthologs. In the case of OPR, the gene *AtOPR3* encodes for the major OPR involved in jasmonic acid biosynthesis (Stintzi and Browse, 2000; Wasternack and Song, 2017) for which we found 3 *Gh* genes coding for *Gh*OPR3 protein orthologs (Figure 4B). In the case of ACX, we found 2 *Gh* genes coding for orthologous proteins of the main ACX of *Arabidopsis*, AtACX1 (Schilmiller et al., 2007). Ultimately, the JASMONIC ACID RESISTANT 1 (AtJAR1) enzyme conjugates jasmonic acid to isoleucine to produce the biologically active conjugated form of jasmonic acid (Staswick and Tiryaki, 2004); the *AtJAR1* gene is induced in response to both pathogen attack and herbivory (Suza and Staswick, 2008; Wasternack and Song, 2017). We found four *Gh* genes coding for *Gh*JAR1 protein orthologs. Analysis of the expression of *Gh* genes coding for orthologs of *Arabidopsis* key enzymes involved in biosynthesis of jasmonic acid revealed that expression of these genes is induced in SJ2 and WMJJ and slightly repressed in NemX in response to RKN-infection (Figure 4B). Most remarkably, the expression of these genes under control conditions and RKN-infection is overall higher in NemX and WMJJ than in RKN-susceptible SJ2 (Figure 4C). Analysis of *Gh* genes coding for orthologs of the jasmonic acid synthesis pathway in *Arabidopsis* corroborated the coordinated upregulation of *Gh* genes coding for multiple enzymes involved in jasmonic acid synthesis in RKN-resistant NemX and WMJJ, with respect to SJ2. To determine whether the higher transcript level of genes involved in jasmonic acid synthesis in NemX with respect to SJ2 is reflected in a difference in the accumulation of this hormone between the two genotypes, we determined the basal level of jasmonic acid in SJ2 and NemX in non-inoculated cotton plants using liquid chromatography coupled to mass spectrometry (UHPLC–MS; Figure 4D). UHPLC–MS quantification revealed that NemX plants accumulate 7.01 pM per g of dry weight of jasmonic acid, two times higher than the basal level present in SJ2 plants (2.83 pM per g of dry weight; Figure 4D). These data support the notion that jasmonic acid is involved in the RKN-resistant response in NemX plants.

**Figure 5:**
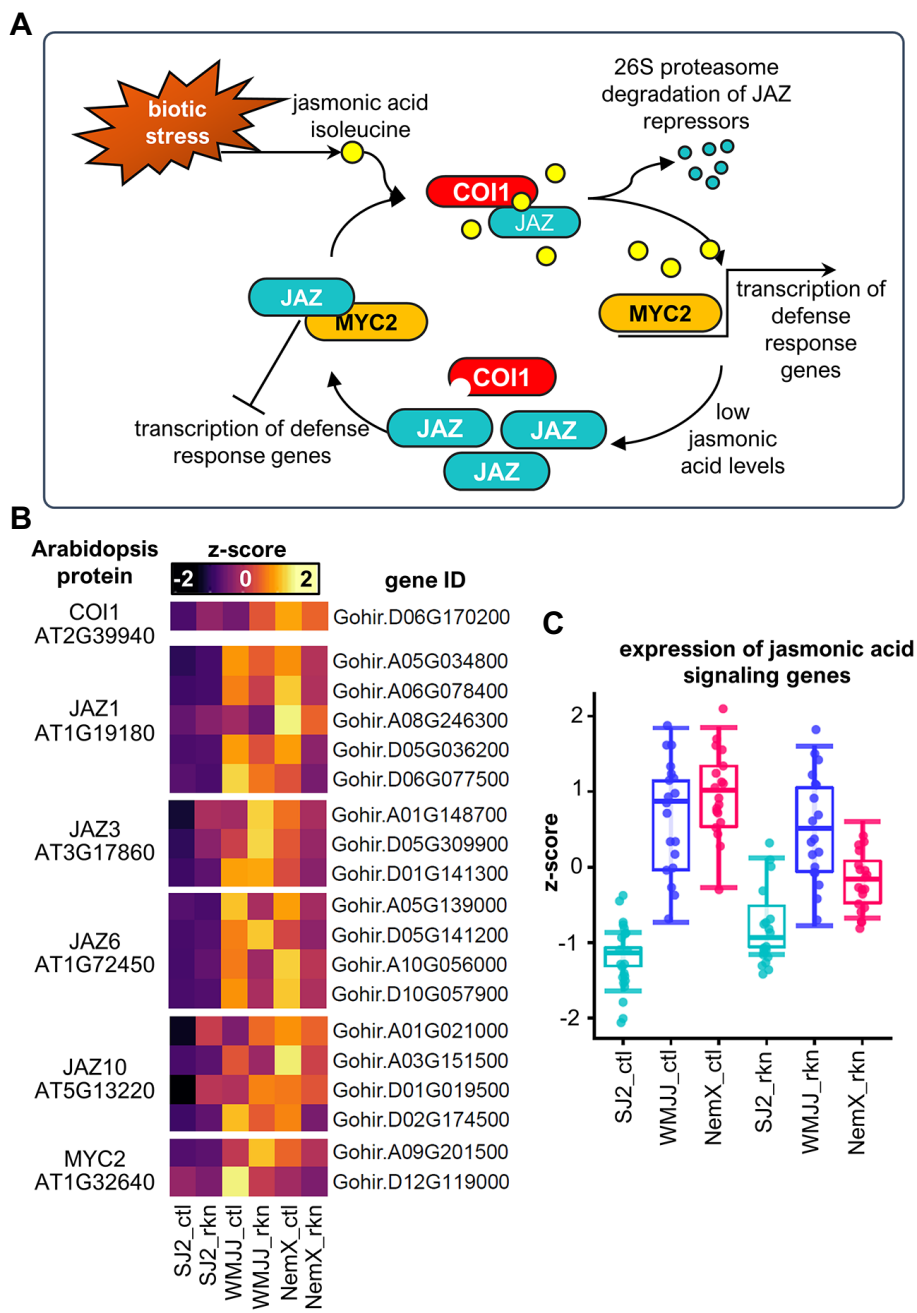
Basal expression of jasmonic acid signaling is upregulated in NemX and WMJJ with respect to SJ2. **(A)** Jasmonic acid signaling in Arabidopsis. **(B)** Heatmap of the expression of *Gh* genes coding for orthologous proteins of Arabidopsis. **(C)** Boxplot analysis of the expression levels of jasmonic acid signaling genes presented in **(B)**.

In plants under biotic stress, jasmonic acid is perceived by the SCF^COI1^-JAZ co-receptor complex (Katsir et al., 2008; Sheard et al., 2010). In this sensing mechanism, JASMONATE-ZIM DOMAIN (JAZ) proteins, which are transcriptional repressors of the jasmonic acid response (Chini et al., 2007), are targeted for ubiquitin-proteasome mediated degradation in the presence of jasmonic-acid-isoleucine by the F-box protein CORONATINE INSENSITIVE 1 (COI1; Sheard et al., 2010; Figure 5A). JAZ proteins are known to be repressors of the MYC2 transcription factor (Chini et al., 2007; Fernández-Calvo et al., 2011) which activates jasmonate-dependent plant defense responses to wounding and pathogen attack (Dombrecht et al., 2007; Pozo et al., 2008). There is a negative-regulation feedback loop between MYC2 and JAZ proteins because the transcription of multiple JAZ proteins is activated by MYC2 (Chini et al., 2007). Therefore, the plant cell can respond properly to jasmonic-acid-isoleucine fluctuations by accumulating and degrading JAZ proteins which will turn off the response to jasmonic acid rapidly in the absence of this signaling molecule (Figure 5A). To further characterize the upregulation of jasmonic acid signaling in RKN-resistant genotypes with respect to RKN-susceptible SJ2, we determined the genes that code for *Gh* protein orthologs of *Arabidopsis* enzymes involved in jasmonic sensing and signaling for those that are more expressed in NemX and WMJJ than in SJ2 (Figure 5B). We found one *Gh* gene coding for a cotton protein ortholog of the F-box jasmonic acid co-receptor AtCOI1. The F-box AtCOI1 interacts with AtJAZ1,3,6,10 co-receptor proteins (Pauwels and Goossens, 2011) for which we found 6, 3, 4 and 4 *Gh* genes coding for AtJAZ1,3,6,10 orthologous proteins, respectively. In the case of the MYC2 transcription factor we found 2 *Gh* genes that code for AtMYC2 orthologous proteins (Figure 5B). Overall, expression data from *Gh* genes encoding orthologs of jasmonic acid signaling in *Arabidopsis* indicate enhanced expression of this gene set in resistant WMJJ and NemX in comparison with susceptible SJ2 (Figure 5C).

### Basal Expression of Salicylic Acid Signaling Genes Is Upregulated in NemX, and in WMJJ to an Intermediate Extent, With Respect to SJ2

**Figure 6:**
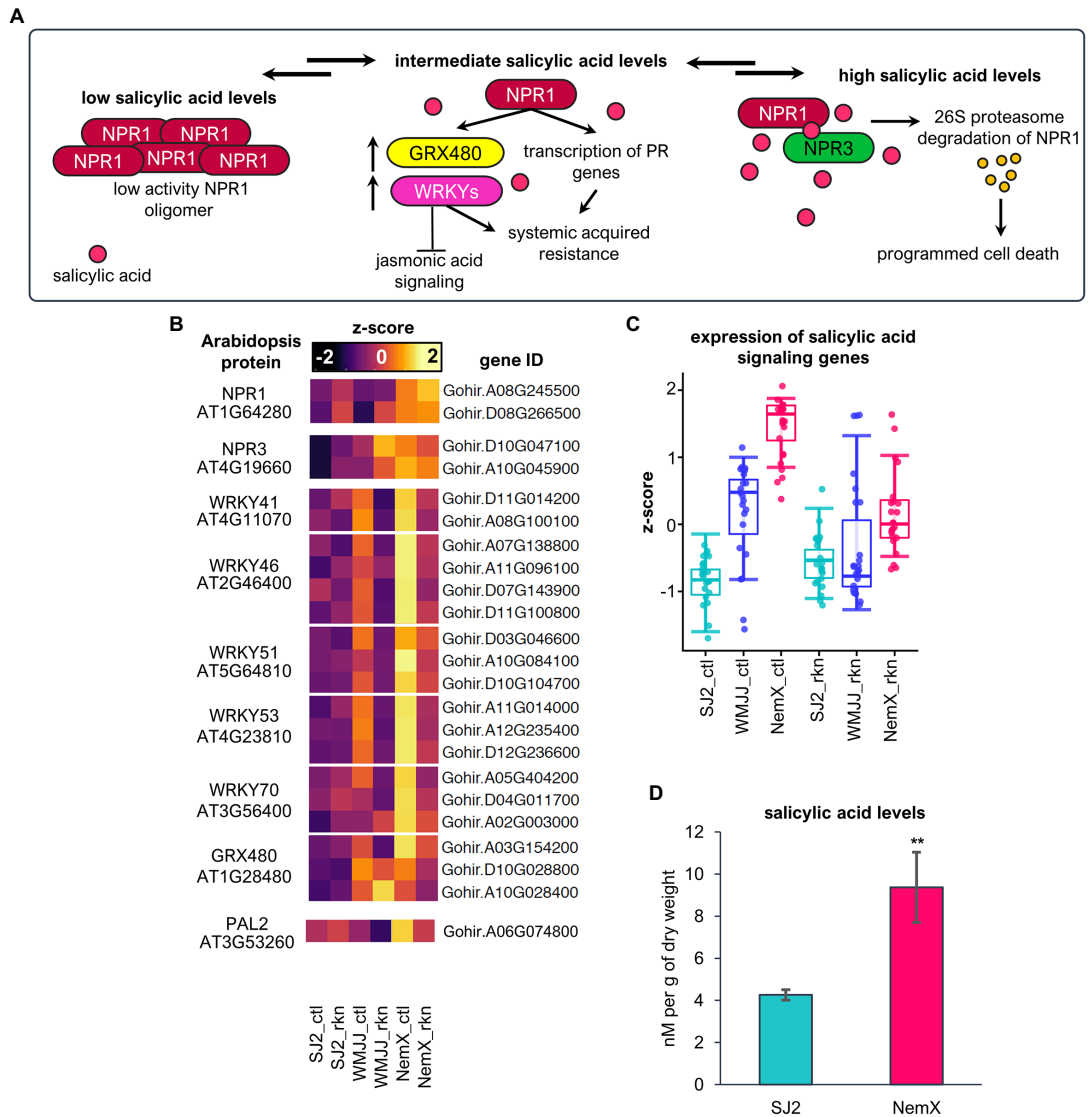
Basal expression of salicylic acid signaling genes is upregulated in NemX and in WMJJ to an intermediate extent, with respect to SJ2. **(A)** Salicylic acid signaling in Arabidopsis. **(B)** Heatmap of the relative expression of *Gh* genes coding for orthologous proteins of Arabidopsis. **(C)** Boxplot analysis of the expression levels of salicylic acid synthesis genes presented in **(B)**. **(D)** Quantification of salicylic acid in SJ2 and NemX cotton plants using UHPLC coupled to MS. Salicylic acid levels were determined in nanomoles (nM) per gram of dry weight. Student *t*-test was performed to determine statistically significant differences (*n* = 3) which are indicated with two asterisks ( *p* < 0.01).

Previously, we observed that salicylic acid responses are enriched in the set of genes with enhanced expression in NemX, but not in WMJJ (Figure 3F). Therefore, to get insights into the regulation of the salicylic acid response and its contribution to RKN-resistance in the tested cotton cultivars, we also decided to analyze the expression of genes that code for *Gh* orthologous proteins of the salicylic acid response in *Arabidopsis* (Figure 6). The transcription co-factor NON-EXPRESSOR OF PATHOGENESIS RELATED 1 (NPR1) is the master transcriptional regulator of salicylic acid induced transcriptional responses and controls the activation of systemic acquired resistance (SAR) in plants in response to salicylic acid levels, conferring immunity to a wide diversity of pathogens (Cao et al., 1997; Backer et al., 2019). In the presence of low salicylic acid levels, NPR1 is present in the cytoplasm as a mostly inactive oligomer (Figure 6A), however, when salicylic acid levels increase to an intermediate level, the redox fluctuation of the cell induces NPR1 activation through its monomerization and translocation to the nucleus (see Fu and Dong, 2013 for a review on NPR1 regulation; Figure 6A). To increase NPR1 activity, NPR1 stimulates the transcription of WRKY transcriptional regulators (for example AtWRKY41,46,51,53,70) that activate defense responses related to systemic acquired resistance and, at the same time, these defense-responses are involved in the downregulation transcription of jasmonic-acid-responsive genes (Wang et al., 2006b; Caarls et al., 2015). Moreover, the induction of *GRX480*, a glutaredoxin that stimulates transcription of salicylic acid response genes and repression of jasmonic acid signaling is dependent on a functional NPR1 (Ndamukong et al., 2007). Under high salicylic acid conditions NPR1 turnover is accelerated by NPR3/4-dependent ubiquitylation and further degradation occurred by the 26 s proteasome which triggers programmed cell death (Fu et al., 2012; Figure 6A). Remarkably, we found out that the expression of two genes coding for *Gh* homolog proteins of AtNPR1 is higher in NemX than in WMJJ and SJ2 (Figure 6B). Furthermore, we also found that the expression of genes coding salicylic acid co-receptor *Gh*NPR3 proteins is also higher in resistant NemX. These data indicate enhanced salicylic acid signaling in the NemX cultivar. Moreover, the expression of *Gh* genes coding for WRKY transcription factors and *Gh*GRX480, which are activated by salicylic acid and repress the jasmonic-acid response, are enhanced in NemX under non-infected control conditions and repressed in response to RKN-infection (Figure 6B). We also found that the expression of an *Arabidopsis* gene that codes for an homolog of *PHENYLALANINE AMMONIA-LYASE 2 (AtPAL2)*, which is involved in early steps of the salicylic acid synthesis pathway (Huang et al., 2010), is upregulated in NemX with respect to WMJJ and SJ2 (Figure 6B), which correlated with a constitutive expression of salicylic acid signaling genes in NemX with respect to both WMJJ and SJ2 (Figure 6C). High basal levels of salicylic acid in NemX plants, prior to RKN infection, might explain the upregulation of salicylic acid signaling genes and a higher degree of RKN resistance. Thus, we sought to determine the levels of this phytohormone in SJ2 and NemX cotton plants using UHPLC–MS analysis (Figure 6D). UHPLC–MS quantification revealed that NemX plants have a basal level of 9.36 nM per g of dry weight, more than two times the basal level of salicylic acid present in SJ2 plants (4.25 nM per g of dry weight; Figure 6D). These data correlate with the basal transcriptional upregulation of salicylic acid signaling observed in NemX with respect to SJ2.

### Transcriptional Profiling Revealed Enhanced Expression of PAMP Receptor Kinases and TIR-NBS-LRR Protein Kinases Related to Nematode Resistance in NemX

**Figure 7:**
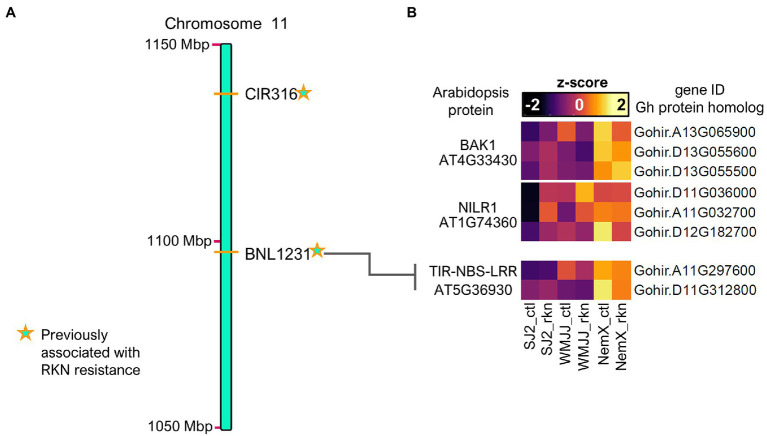
Basal expression of genes coding for PAMP-receptors and Resistance proteins is enhanced in NemX with respect to WMJJ and SJ2. **(A)** Section of linkage map illustrating microsatellite markers previously associated with RKN-resistance (Wang et al., 2020) present in *Gh* chromosome 11. **(B)** Heatmap of expression values of *Gh* genes coding for orthologs of (top) BAK1 a co-receptor for PAMPs, (middle) NILR1 a kinase required for innate immunity to nematodes in Arabidopsis and (bottom) a TIR-NBS-LRR R protein possibly implicated in nematode resistance in cotton.

Plant defense responses can be broadly dissected into two layers of immunity. The first layer is activated through the recognition of pathogen-associated molecular patterns (PAMP) and is known as PAMP-triggered immunity (PTI). PTI comprises the activation of several defense responses which can include the activation of oxidative burst, kinase-dependent signaling cascades and the activation of defense-related gene expression (Macho and Zipfel, 2014). PTI is under the control of surface-localized pattern recognition receptor (PRR) proteins which contain a transmembrane domain with an external domain capable of ligand binding. In plants, PRRs are either receptor-like kinases (RLKs) or receptor like proteins (RLPs; Macho and Zipfel, 2014). An example of RLK is found in the *Arabidopsis* leucine-rich receptor (LRR) AtFLS2 which forms a signaling heterodimer with the protein BRI1 ASSOCIATED RECEPTOR KINASE 1 (BAK1) upon bacterial flagellin perception and activates PTI (Sun et al., 2013). Nematodes are known to induce PTI in *Arabidopsis*, and data indicates that the BAK1 co-receptor is involved in this response as *bak1-5*, and mutants have enhanced susceptibility to RKN (Teixeira et al., 2016). In the case of PTI in response to plant-parasitic nematode infection, only one RLK that is essential for nematode-resistance in *Arabidopsis* has been described and is encoded by the gene *NEMATODE INDUCED LRR-RLK 1* (*NILR1*; Mendy et al., 2017). Interestingly, we found enhanced expression of *Gh* genes coding for homologs of AtBAK1 and AtNILR1 in resistant NemX in comparison with both WMJJ and SJ2 (Figure 7).

A second layer of immunity is activated by pathogen effector molecules which are directly delivered into the plant cell and is known as effector triggered immunity (ETI; for review see Spoel and Dong, 2012). ETI is activated by cytoplasmic receptor proteins with a nucleotide binding site and a leucine rich receptor domain known as TIR-NBL-LRR proteins which are also known as resistance (R) proteins. R proteins are known to activate the local hypersensitive response which can result in systemic activation of plant immune responses *via* systemic acquired resistance (Spoel and Dong, 2012). Because plant-parasitic nematodes like RKN pierce plant cells and secrete effectors, ETI is also triggered in response to nematode infection (Sato et al., 2019). In our previous genetic and QTL mapping studies, we first reported the characterization of the RKN-resistance region and located a rkn1 factor and a QTL that contributes to RKN-resistance on chromosomes A11 and D11 in NemX (Wang et al., 2006a, 2020; Ulloa et al., 2010). Interestingly, we identified sequences coding for TIR-NBL-LRR R proteins which are candidates for RKN-resistance in this region. To further identify the genes coding for R proteins in the NemX genome, we performed a blast of these resistance sequences (Wang et al., 2020) against the TM-1 genome (see section “Materials and Methods”) and obtained 121 and 144 genes hits belonging to the A11 (11) and D11 (21) chromosomes, respectively (Supplementary Figure S3; Supplementary Material). To test whether some of these candidate genes have higher expression in resistant NemX, we performed Venn analysis of the blast hits with the genes that have higher basal expression in NemX with respect to SJ2 (Supplementary Figure S3). Because RKN-resistance in WMJJ has been mapped to chromosome 14 (Gutiérrez et al., 2010), we decided to reduce the list of hits by deleting those that have enhanced expression in WMJJ, which are most likely not related to NemX-related RKN resistance (Supplementary Figure S3). Only two genes, *Gohir.A11G297600* and *Gohir.D11G312800*, remained in our list after filtering for chromosomes 11 and 21. Most remarkably, these two genes code for *Gh* protein homologs of the same *Arabidopsis* R protein TIR-NBS-LRR (AT5G36930). Our results indicate that these genes coding for a *Gh* TIR-NBS-LRR have enhanced expression in NemX with respect to WMJJ and SJ2 and, therefore, are very promising gene candidates responsible for RKN-resistance in NemX (Figure 7).

## Discussion

Although RKN is a major threat to cotton production worldwide, very little is known about the molecular mechanisms of RKN-resistance in cotton. To address this problem, we performed whole-genome transcriptomic profiling of RKN-resistance in Upland cotton cultivars by harnessing the recently published *G. hirsutum* genome sequence (Chen et al., 2020) and using it as a reference genome to characterize the transcriptional landscape of the roots of RKN-resistance cultivars Acala NemX and WMJJ (Wang et al., 2006a; Gutiérrez et al., 2010) and the susceptible Acala SJ2 (Wang et al., 2006a). Gene expression profiling revealed that the control and RKN-infected NemX RNA-seq libraries cluster with the SJ2 libraries from RKN-infested roots (Figure 2A). These results suggest a constitutive RKN-infested-like transcriptional behavior in non-infected NemX roots. Moreover, comparative analysis of differential gene expression data suggests that RKN-susceptibility positively correlates with the extent of the transcriptional response of each cultivar to RKN-infestation which has been observed previously for RKN-resistant cultivars of tomato (Schaff et al., 2007) and alfalfa (Postnikova et al., 2015). Subsequent GO analysis revealed that functional categories related to the hormonal regulation plant immunity, are enriched in the transcriptional response to RKN-infestation of WMJJ and SJ2 but not in the transcriptional response of NemX (Figure 2D). This result was unexpected as one of the possibilities that could explain NemX RKN-resistance is an increased immune response which might be boosted by an enhanced transcription of genes involved in the synthesis of salicylic and jasmonic acid, as well as the signaling pathways activated by these two plant hormones. However, further differential expression analysis coupled to GO functional characterization of the genes that have higher basal expression in NemX revealed that the transcriptional regulation of the synthesis and signaling pathways of salicylic and jasmonic acid were constitutively activated in NemX and were little responsive to RKN infection (Figures 4–6). These results agree with a RKN-infested-like transcriptional behavior naturally present in the resistant NemX cultivar.

Expression analysis of *Gh* genes coding for orthologs of *Arabidopsis* proteins involved in jasmonate biosynthesis revealed that these genes are more expressed in RKN-resistant cultivars WMJJ and NemX than in susceptible SJ2. This transcriptional enhancement of jasmonic acid biosynthesis could indeed explain at least in part the RKN resistance as exogenous application of jasmonic acid to oats, spinach and RKN-susceptible cultivars of tomato activates defense responses that reduce nematode reproduction and confer nematode resistance (Soriano et al., 2004, 2007; Cooper et al., 2005). Data shows that the expression of key jasmonic acid sensing and signaling pathways is enhanced in the roots of NemX and WMJJ with respect to SJ2 (Figures 5B,C). This was expected because the putative increase in jasmonic acid synthesis most likely leads to increased activation of jasmonic-acid-responsive genes in RKN-resistant NemX and WMJJ. The interplay between jasmonic acid and salicylic acid is known to modulate plant immunity to either necrotrophic or biotrophic pathogens (Pieterse et al., 2012). However, as is the case of several plant pathogens, plant-parasitic nematodes like *M. incognita* can display both lifestyles which might be the reason why plants deploy both jasmonic and salicylic acid signaling-dependent defense responses to cope with RKN-infestation (Goverse and Bird, 2011; Martínez-Medina et al., 2017). GO enrichment analysis revealed that salicylic acid responses are enriched in the set of genes with enhanced expression in NemX, but not in WMJJ (Figure 3F). The mechanisms of resistance operating in WMJJ suppress nematode egg deposition (Gutiérrez et al., 2010), while in NemX both egg deposition and root galling are suppressed (Wang et al., 2006a), which probably enables NemX a higher RKN-resistance than WMJJ. It is most likely that these differences in RKN-resistance are explained by the fact that the expression of both salicylic- and jasmonic acid-responsive genes is enhanced in NemX, whereas in WMJJ only jasmonic acid responsive genes is enhanced (Figures 3E,F; 5C).

**Figure 8:**
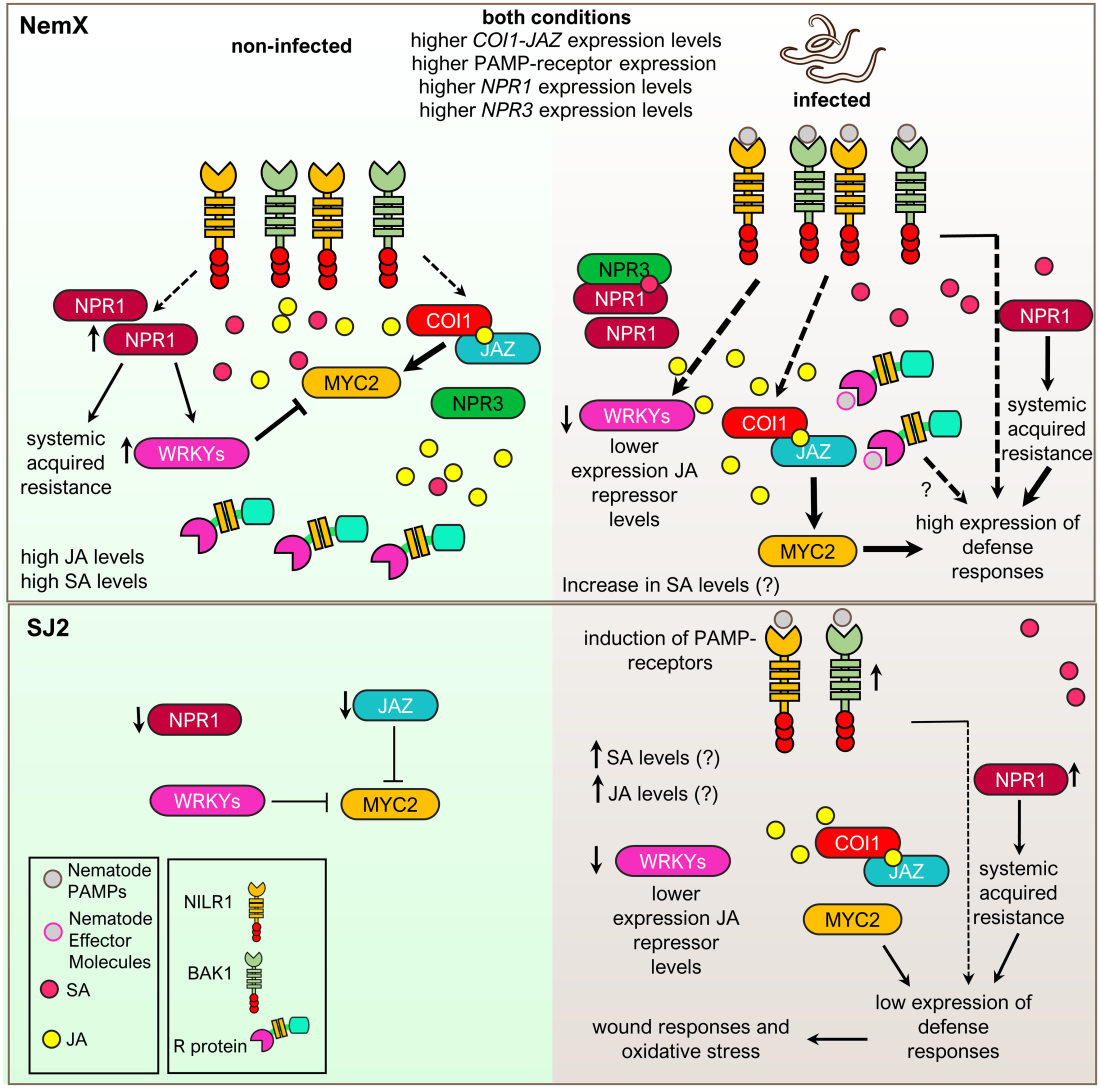
Putative model that explains RKN-resistance in the NemX cultivar at the molecular level. The model depicts putative molecular mechanisms that occur in RKN-resistant NemX (upper panel) and RKN-susceptible SJ2 (lower panel) in response to RKN-infection (right). NemX presents higher basal levels of salicylic (SA) and jasmonic acid (JA) defense-related phytohormones than SJ2. Enhanced expression of the salicylic acid master regulator *NPR1* and the salicylic acid co-receptor *NPR3* results in enhanced expression of genes related to systemic acquired resistance. Enhancement of *WRKY* expression, most likely caused by enhanced basal levels of salicylic acid and NPR1 signaling, antagonizes jasmonic acid signaling but the enhanced basal levels of jasmonic acid and the expression of jasmonic acid signaling components remain high which might protect the plant in the case of RKN infection. It is likely that this constitutive expression of jasmonic and salicylic acid signaling genes is caused by enhanced basal levels of these phytohormones and the constitutive expression of RLKs and R proteins in NemX. In SJ2, this mechanism of resistance is not present, and the root presents lower basal expression of defense signaling genes. In NemX, RLKs and R proteins have a higher expression level and likely detect nematode pathogen associated molecular patterns (PAMPs) and nematode effector molecules, respectively, to trigger high levels of defense transcriptional responses which constitutively prevent RKN invasion and egg deposition. In the case of SJ2, RLK and R protein receptors have a lower basal level expression and are mildly induced to a lower level than in NemX in response to RKN infection which correlates with lower levels of expression of defense-related genes which results in a more successful RKN infection in SJ2 than in NemX. The successful RKN-infestation in SJ2 leads to the expression of wound and oxidative stress responses related to cellular death and damage.

Interestingly, salicylic acid works as a trigger of defense priming, a plant adaptation that prepares plants to respond rapidly and strongly to pathogen challenges after an initial stimulus and usually involves the prophylactic activation of systemic acquired resistance-related genes (Mauch-Mani et al., 2017). In the case of tomato, biotic stimulus by *Trichoderma* spp. activates the priming of salicylic acid defense responses and inhibits RKN root invasion and a shift from salicylic acid to jasmonic acid signaling in the feeding stage of nematode infection, also triggered by Trichoderma *spp.*, inhibiting root galling and compromising nematode fertility (Martínez-Medina et al., 2017). The shift from salicylic acid to jasmonic acid signaling might operate in NemX at the molecular level to inhibit RKN. However, data presented in Figures 4, 7 indicate that there is a constitutive rather than rapid and strong activation of responses characteristic of priming. This indicates, therefore, that the RKN-resistance mechanism in NemX does not operate in priming-like fashion to activate the increase of gene expression in response to RKN-infection (Figure 8), but rather a constitutive expression of defense response genes. Moreover, enhanced expression of the genes encoding for the salicylic and jasmonic acid receptor components NPR1/3 and COI-JAZ and higher levels of salicylic acid and jasmonic acid in NemX plants than in the susceptible SJ2, suggest that resistance in NemX might be activated by the constitutive activation of these two hormone-signaling pathways. The underlying molecular regulator, or regulators, behind the upregulation of jasmonic and salicylic acid synthesis and signaling in NemX remains an interesting research perspective.

Enhanced expression of jasmonic acid and salicylic acid receptors most likely enables NemX to rapidly activate defense responses upon nematode challenge (Figure 8). The constitutive activation of jasmonic-acid-dependent defense responses implicates fitness tradeoffs that impose a penalty on plant development and yield (Züst and Agrawal, 2017). Interestingly, the RKN-resistance mechanism present in NemX does not seem to present a significant toll on plant fitness, as NemX cotton presents normal and robust growth with only a slight lint yield reduction when compared to other cotton entries under non-infected field conditions (Ogallo et al., 1997). As previously mentioned, WRKY transcription factors are enhanced by salicylic acid signaling and antagonize jasmonic acid signaling in plants (Caarls et al., 2015). These transcriptional regulators present enhanced expression in NemX (Figure 6) which is most likely caused by enhanced salicylic acid signaling in this genotype. Therefore, an interesting hypothesis is that enhanced salicylic acid signaling enables NemX plants to maintain high expression levels of jasmonic acid signaling components while avoiding the fitness costs of constitutive expression of jasmonic-acid-dependent defense responses. This could be mediated *via* salicylic acid-inhibition of some of these responses (Figure 8). The specific molecular interactions and signaling mechanisms that underlie the establishment of the RKN resistance mechanism in NemX remain an interesting research perspective to engineer and transfer this resistance mechanism to other crops while avoiding overall fitness and yield costs.

Because both TIR-NBS-LRR proteins and PAMP-RLKs are closely related to the activation of defense responses in plants (Spoel and Dong, 2012; Macho and Zipfel, 2014), enhanced expression of these receptors might be involved in triggering the enhanced and rapid defense signaling in response to nematode challenge in NemX (Figure 8). Moreover, the mechanisms underlying enhanced expression of TIR-NBS-LRR proteins and PAMP-RLKs in NemX remain unknown and are also an interesting topic for future research. Allele variation analysis among RKN-susceptible and resistant cultivars might prove useful to resolve the latter perspective.

Information on resistant and susceptible-infected and non-infected control gene expression profiles and molecular mechanisms of RKN-resistance in cotton is currently very limited. A recent cotton comparative transcriptomic study (Kumar et al., 2019) of RKN susceptible ‘Coker 201’ and resistant ‘M120 RNR’ genotypes at two RKN infection times (12 and 30 days after RKN inoculation) revealed similar results for the transcriptional response of RKN-susceptible cultivars to nematode infection in which high numbers of genes were expressed in susceptible vs. resistant cotton genotypes which agrees with our results and previous results observed in tomato (Schaff et al., 2007) and alfalfa (Postnikova et al., 2015; Figure 2). The GO analysis of the cotton root transcriptional response to RKN-treatment reported by Kumar et al., 2019 revealed enrichment of functional categories such as ethylene signaling, transcription factor activity, hormone regulation of plant immunity and transcriptional defense responses which agree with our results (Figure 2D).

Previous RKN genetic and QTL mapping studies identified two RKN-resistance QTLs, one in chromosome A11 and the other in D11 in NemX. Within the interval of confidence of the RKN-resistance QTL in A11 we identified two *Gh* genes coding orthologs to *Arabidopsis* AT5G36930 TIR-NBS-LRR, that have enhanced expression in NemX with respect to WMJJ and SJ2. This family of proteins has been shown to underlie RKN-resistance in tomato and potato (Williamson and Kumar, 2006). The enhanced TIR-NBS-LRR R gene expression confirmed that the genomic region close to the microsatellite marker BNL1231 in chromosome 11 is indeed associated with RKN resistance (Wang et al., 2020). Interestingly, no enhanced expression was detected in CC-NB-LRR R genes which localizes in the rkn1-tightly-linked region close to the microsatellite marker CIR316, located also in chromosome 11, with a 10 cM genetic distance from BNL1231. This indicates that additional mechanisms might be contributing to the phenotypic variation of RKN resistance in the NemX-CIR316 region present in chromosome 11 (Wang et al., 2006a, 2020). The TIR-NBS-LRR genes are very promising candidates for RKN-resistance in NemX (Figure 7), which may increase the efficiency of marker assisted selection in cotton breeding programs. Further functional characterization experiments might enlighten the precise molecular mechanisms by which the expression of the *GhNILR1* and *GhTIR-NBS-LRR* genes, or their function, is enhanced in NemX and should provide insights into mobilization of NemX-RKN-resistance to other cotton varieties of commercial interest.

## Data Availability Statement

The datasets presented in this study can be found in online repositories. The names of the repository/repositories and accession number(s) can be found at: National Center for Biotechnology Information (NCBI) Gene Expression Omnibus (GEO) database under accession number GSE190503.

## Author Contributions

MU conceived the idea, organized and planned experiments, analyzed data and edited the manuscript. DL-A analyzed data, supervised bioinformatic work, and wrote paper together with JOO-R and LH-E. LH-E analyzed data, helped designed figures and wrote paper together with JOO-R and DL-A. JOO-R performed RNA-seq data analysis, designed figures and wrote the first draft of the manuscript. PR designed and performed RKN experiments, reviewed and edited the manuscript. CW performed RKN-infection experiments and collected root tissue. PK prepared library and carried out Illumina RNA-sequencing. PP analyzed data and edited the manuscript. H-RN-G performed UHPLC–MS analysis of cotton tissue. All authors contributed to the article and approved the submitted version.

## Funding

Partial support for this work was provided by grants from USDA-ARS (grant 3096-21000-022-00-D to MU and PP), Cotton Incorporated Cary, NC (grant 21-844 to DL-A), and USDA-ARS-NACA (grant 21A546 to DL-A), and the State of Texas Governor’s University Research Initiative (GURI)/Texas Tech University (grant 05-2018 to LH-E).

## Conflict of Interest

The authors declare that the research was conducted in the absence of any commercial or financial relationships that could be construed as a potential conflict of interest.

## Publisher’s Note

All claims expressed in this article are solely those of the authors and do not necessarily represent those of their affiliated organizations, or those of the publisher, the editors and the reviewers. Any product that may be evaluated in this article, or claim that may be made by its manufacturer, is not guaranteed or endorsed by the publisher.

## Footnotes

^1^ https://www.bioinformatics.babraham.ac.uk/projects/fastqc/

^2^ www.cottongen.com

## Supplementary Material

Supplementary Figures PDF file containing supplementary figures cited in the text.

Supplementary Dataset Excel file containing RNA-seq supplementary data.
