# Supplementary material for: Root-Knot Nematode Resistance in Gossypium hirsutum Determined by a Constitutive Defense-Response Transcriptional Program Avoiding a Fitness Penalty
Source: Front Plant Sci. 2022 Apr 13;13:858313. doi: 10.3389/fpls.2022.858313 (PMC9044970; doi:10.3389/fpls.2022.858313)
Supplement: Supplementary Figures — PDF file containing supplementary figures cited in the text. [file Data_Sheet_1.zip › Supplementary Figures.PDF]

downregulated genes in response to RKN infestation

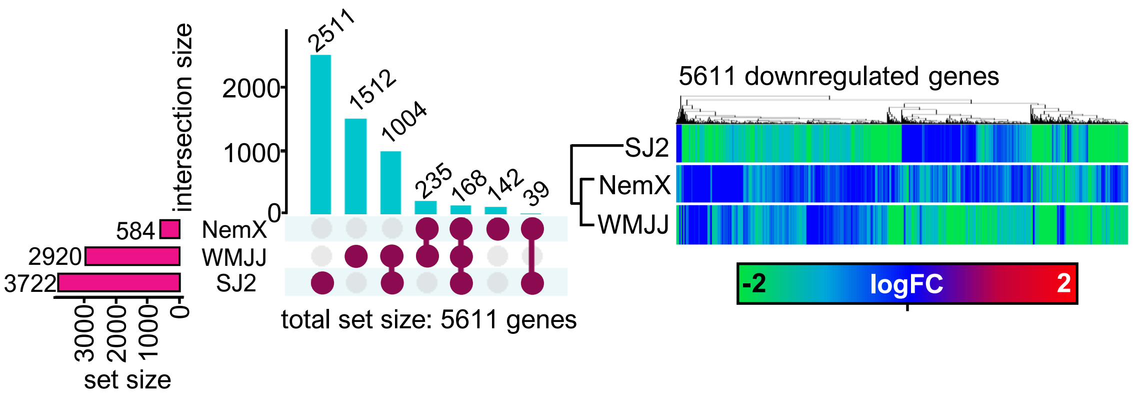

Supplementary Figure 1. Expression profiling analysis revealed a differential downregulation of genes in response to RKN-treatment among cotton accessions SJ2, WMJJ and NemX.

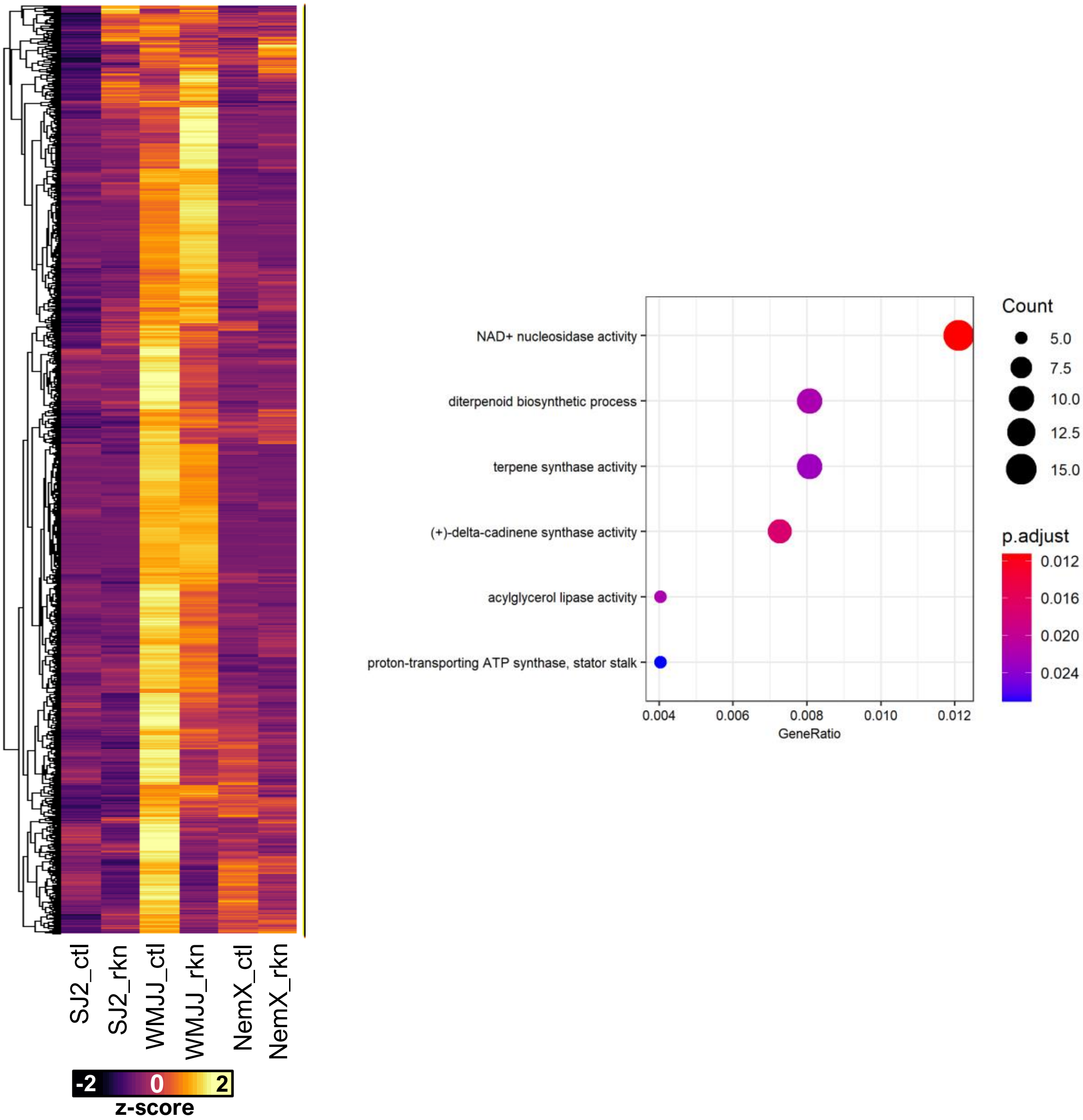

Supplementary Figure 2. Genes with upregulated basal expression in WMJJ with respect to SJ2. (A) Heatmap of the expression of the 1499 genes with upregulated expression in WMJJ with respect to SJ2. (B) Gene Ontology enrichment analysis of functional categories in the set of 1499 genes that present upregulated expression in WMJJ with respect to SJ2.

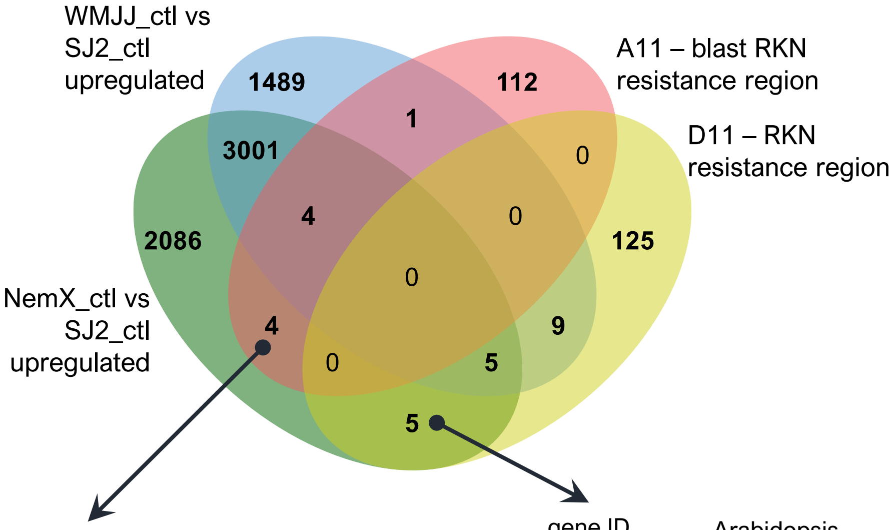

gene ID<br>Gh protein homolog	Arabidopsis protein
Gohir.A11G292500	AT2G34930
Gohir.A11G109300	AT2G16760
Gohir.A11G297600	AT5G36930
Gohir.A11G295000	AT1G09070

gene ID<br>Gh protein homolog	Arabidopsis protein
Gohir.D11G347500	AT3G54100
Gohir.D11G312800	AT5G36930
Gohir.D11G321900	AT5G05140
Gohir.D11G333000	AT3G11670
Gohir.D11G312368	

Supplementary Figure 3. Venn analysis of the blast hits to the rkn1 resistance region in NemX with the genes that have more basal expression in NemX with respect to SJ2.
